# Urea induces intercellular adhesion molecule-1 expression

**DOI:** 10.1186/1471-2210-11-S2-A34

**Published:** 2011-09-05

**Authors:** Dalia El-Gamal, Michael Holzer, Veronika Binder, Sabine Dirnberger, Ákos Heinemann, Gunther Marsche

**Affiliations:** 1 Institute of Experimental and Clinical Pharmacology, Medical University of Graz, 8010 Graz, Austria

## Background

The dramatically increased cardiovascular risk in patients with chronic renal disease cannot be explained entirely by traditional cardiovascular risk factors. The continuous exposure of renal patients to elevated levels of urea may contribute to increased inflammation and oxidative stress. While urea may not be directly toxic, many molecules can be carbamylated through cyanate, a reactive decomposition product of urea. Cyanate irreversibly transforms lysine to ε-amino-carbamyl lysine. This pathway may be of particular relevance since clinical studies have shown that carbamylated proteins are independent risk factors for the development of coronary artery disease and stroke.

## Methods and results

Here we show that urea potently induces intracellular cell adhesion molecule-1 (ICAM-1) expression with subsequently enhanced neutrophil adhesion in human coronary artery endothelial cells. ICAM-1 expression is triggered through a mechanism depending upon activation of the mitogen-activated protein kinase p38 and nuclear factor κB. Interestingly, ICAM-1 expression was not induced when low-molecular weight substances were removed from cell culture medium, ruling out a role of carbamylated (lipo)proteins in ICAM-1 induction. Moreover, in end-stage renal disease patients the extent of plasma protein carbamylation (a marker for cyanate formation) correlated significantly with plasma levels of soluble ICAM-1.

## Conclusions

Collectively, our data raise the possibility that urea amplifies vascular inflammation in renal patients.

